# The establishment of immune infiltration based novel recurrence predicting nomogram in prostate cancer

**DOI:** 10.1002/cam4.2433

**Published:** 2019-07-29

**Authors:** Jialin Meng, Yi Liu, Shiyang Guan, Song Fan, Jun Zhou, Meng Zhang, Chaozhao Liang

**Affiliations:** ^1^ Department of Urology The First Affiliated Hospital of Anhui Medical University Hefei China; ^2^ Institute of Urology Anhui Medical University Hefei China; ^3^ Anhui Province Key Laboratory of Genitourinary Diseases Anhui Medical University Hefei China; ^4^ Department of Epidemiology and Biostatistics, School of Public Health Anhui Medical University Hefei China

**Keywords:** macrophages, nomogram, prognosis, prostate cancer, recurrence‐free survival, tumor‐infiltrating immune cells

## Abstract

Prostate cancer (PCa), a severe health burden for males, accounts for the second frequent cancer and fifth tumor specific death cancer around the world. Several studies on tumor‐infiltrating immune cells (TIICs) have shown inconsistent and controversial results to PCa. We downloaded a gene expression matrix and clinical information from TCGA, and CIBERSORT was used to identify the proportion of TIICs. Wilcoxon's Sign Rank Test evaluated different gene expression levels in PCa and normal tissues. Kaplan‐Meier curves were used to evaluate the associations of TIICs and recurrence‐free survival (RFS). Finally, based on the preset *P‐value* of .05, the distribution of TIICs in 73 PCa tissues and 11 normal tissues was illustrated. Activated CD4^+^ T cells and M0 macrophages account for a high proportion in PCa tissues, while neutrophils and monocytes were found at a high density in normal tissues. Further results showed that the density of plasma cells, Treg cells and resting mast cells were associated with advanced PCa. Additionally, M2 macrophages affected the RFS of PCa patients, and AR was also involved. In the current study, we first evaluated the immune infiltration among PCa and revealed that M2 macrophages could predict the prognosis of PCa patients. Meanwhile, based on the LASSO regression analysis, we established a novel nomogram to assess the recurrence risk of PCa based on immune cell proportions and clinical features.

## 1 INTRODUCTION

Prostate cancer (PCa) is the second most common cancer and the fifth leading cause of death from cancer in men around the world, with an estimated 307 000 deaths representing 6.6% of total cancer mortality in males.^1^, ^2^, ^3^ The latest data also show that PCa is the most common malignancy and second leading source of death in America, which accounts for 174 650 estimated new cases and 31 620 estimated deaths in 2019.^4^ For Europe, PCa accounts for almost 21.8% of all newly diagnosed cancer patients, along with approximately 10% of cancer deaths. PCa is the first leading type of tumor in 28 European countries, and the second prominent type in another seven countries.^5^ In China, the incidence of PCa has increased sharply, along with the application of ultrasound‐guided prostate biopsy.^6^, ^7^ The progression of PCa is prolonged compared with other tumors, leading to 5‐year survival rates up to greater than 80%.^8^, ^9^, ^10^ However, for advanced PCa, hormonal deprivation therapy can temporarily inhibit tumor progression through the androgen receptor signaling pathway, but this function is only effective for approximately 2 years. Then, serum levels of prostate‐specific antibody (PSA) increase again rapidly, meeting the stage of castration‐resistant prostate cancer (CRPC), which is the leading cause of tumor‐specific death in PCa.^11^, ^12^, ^13^, ^14^

Tumor‐infiltrating immune cells (TIICs) are essential components of the tumor microenvironment and can alter the immune status of the tumor. Several studies have demonstrated therapeutic strategies against tumors by targeting TIICs.^15^, ^16^, ^17^, ^18^, ^19^ Clinical outcomes and the potential mechanisms involving PCa and TIICs have been widely reported. Kaur et al^20^ marked the T cells in PCa tissues with CD3, CD8, and FOXP3 immunostaining and revealed that ERG activity and PTEN loss were affected by the high proportion of T cells, but clinical outcomes showed no association with these results. Petitprez et al^21^ reported that a high proportion of CD8^+^ T cells could lead to a greater risk of advanced development of PCa patients with node metastasis. This effect was accompanied with increased expression of PD‐L1 from tumor cells. Philippe et al^22^ demonstrated that natural killer (NK) cells and macrophages function in androgen deprivation therapy patients and that the abundance of NK cells decreased the risk of reduced tumor progression, but the high proportion of macrophages could increase the risk of biochemical recurrence.

Due to the limitations of methods and techniques, prior studies focused on finite areas of the immune response, whereas the full view of the immune response is based on the infiltration of different TIICs. Alternatively, the immunohistochemistry (IHC)‐based labels of TIICs are not precise, and several TIICs may express the same makers on the membrane, leading to the inaccurate measurements of TIIC density in PCa tumor tissues. In the present study, the proportion of 22 TIICs in PCa tissues was illustrated by a newly developed deconvolution algorithm, CIBERSORT, which can predict the density of TIICs through the expression of 547 key genes, and revealed the potential effect of TIICs in PCa prognosis.

## 2 MATERIALS AND METHODS

### 2.1 Gene expression database

In the current study, TCGA database of PCa tissues and control tissues were enrolled. We downloaded the overall gene expression profiles and clinical information of patients from TCGA up to June 31, 2019. Finally, the gene expression profiles of 499 PCa samples and 52 controls were prepared for subsequent analysis. We performed the stable multi‐array average algorithm to preprocess the recorded raw data. The mRNA sequencing data of TCGA database were first converted to a microarray‐similar result using the voom method (variance modeling at the observational level). Then, we annotated the gene ID, and the mRNA sequencing results were normalized with the “limma” package of R, to average the data of repeated genes and delete results that were not available. In Figure S1, we displayed the details of the study design and the different clinical stages when the samples were recorded.

### 2.2 Inference of immune infiltration in samples

The analytical tool called CIBERSORT was used in the study, which can quantify the percentage of different types of TIICs accurately, under the complex “gene signature matrix” based on 547 genes.^23^ In the current study, we illustrated the immune infiltration of each sample with the LM22 signature file, which can define 22 subtypes of immune cells, including naïve B cells, memory B cells, plasma cells, CD8^+^ T cells, naïve CD4^+^ T cells, resting memory CD4^+^ T cells, activated memory CD4^+^ T cells, follicular helper T cells, regulatory T cells (Treg cells), gamma delta T cells, resting NK cells, activated NK cells, monocytes, M0 macrophages, M1 macrophages, M2 macrophages, resting dendritic cells, activated dendritic cells, resting mast cells, activated mast cells, eosinophils, and neutrophils, with the preset signature matrix at 1000 permutations. After using the CIBERSORT program, the distribution of 22 subtypes of TIICs was presented, along with the results of correlation coefficient, *P*‐value and root mean squared error (RMSE), which can evaluate the accuracy of the results in each sample. The P‐value ≤ .05 reflects a statistical connotation of the results of deconvolution across all cell subsets for each sample and is useful for excluding results with less accuracy. Finally, 73 PCa samples and 11 control samples were selected for later analysis because they met the required P‐value.

### 2.3 Statistical analyses

For each type of TIICs, we assessed the different levels in patients and controls with Wilcoxon's Sign Rank Test and displayed the violin plot. Using the Pearson correlation coefficient, correlations between two immune cell subsets were evaluated. The different distribution of TIICs in several subgroups of clinical features were evaluated with Wilcoxon's Sign Rank Test in parameters divided into two groups and with the Kolmogorov‐Smirnov test in parameters separated into more than two groups. Kaplan‐Meier (K‐M) curves were constructed to analyze and evaluate the associations of immune cell infiltrate and corresponding recurrence‐free survival (RFS) using the log‐rank test. The median of the proportion of each cell type was computed to divide the patients into high‐ and low‐density groups. The nomogram was constructed using the rms package in R (http://www.r-project.org). ROC and AUC were completed to assess the discriminatory ability of the nomogram, while decision curve analysis was also performed to evaluate the usability of the nomogram. STRING, an online tool that could reveal the functional protein association networks, was employed to identify androgen receptor associated proteins.^24^ All analyses were conducted using R version 3.5.3. *P < *.05 was considered statistically significant.

## 3 RESULTS

### 3.1 The distribution of TIICs in PCa tissues and controls

**Figure 1:**
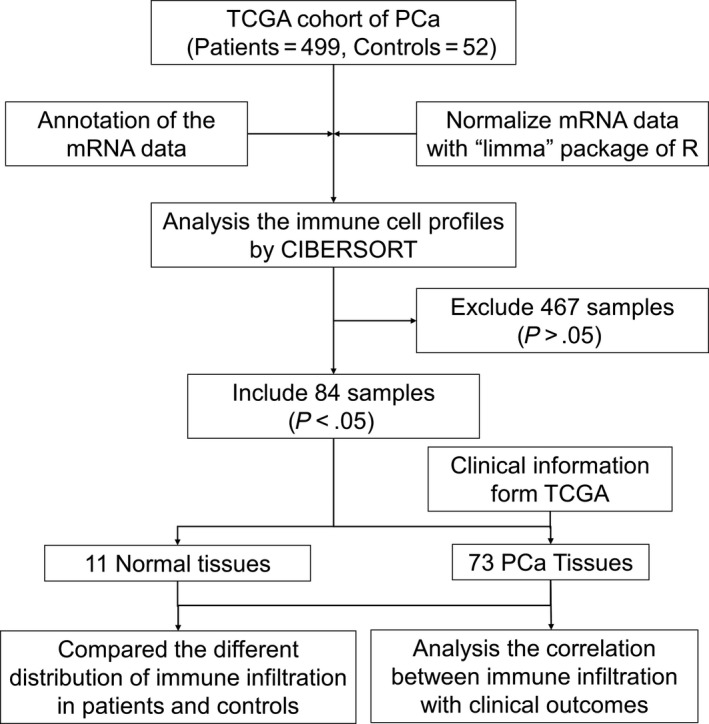
Flowchart detailing the study design and samples at each stage of analysis. CIBERSORT, cell type identification by estimating relative subsets of known RNA transcripts; TGCA, The Cancer Genome Atlas

**Figure 2:**
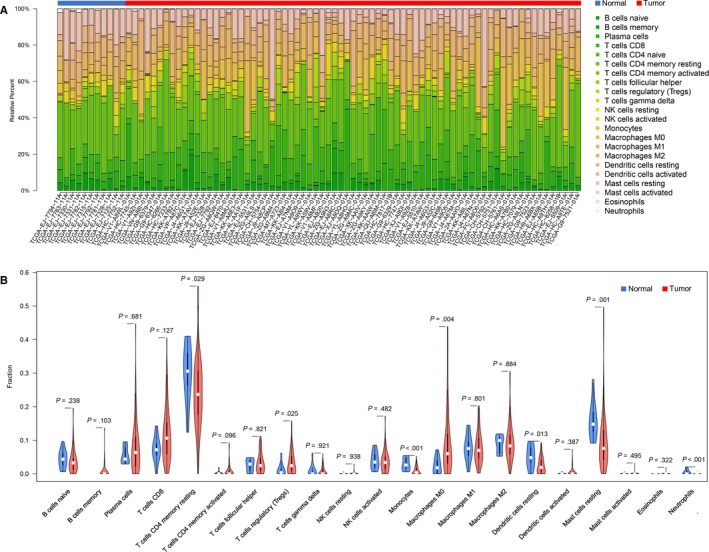
The performance of cell type identification by estimating relative subsets of known RNA transcripts for characterizing tumor‐infiltrating immune cells (TIIC), composition in prostate cancer (PCa), and control tissues. A, The difference of immune infiltration in each sample of PCa and control tissues. B, The quantified contrast of the distribution of TIIC subtypes between PCa and control tissues

The differences between 22 subpopulations of TIICs in PCa tissues and normal cells were investigated using the CIBERSORT algorithm; finally, 11 control tissues and 73 PCa tissues met the preset criteria (*P < *.05). The flowchart of study design and the process of samples enrolled is shown in Figure 1. The results obtained from the tissues of 73 PCa and 11 control tissues are summarized in Figure 2A, and details of the distribution were provided in Table S1. Obviously, the proportion of TIICs in PCa varies significantly between both the intra‐ and intergroup; then, we compared the average distribution of TIICs to assess the gross inconsistencies. We evaluated the average proportion of each immune cell type in PCa tissues and adjacent tissues. The result revealed that resting CD4^+^ memory T cells were highly present in normal tissues (*P* = .029), as well as monocytes (*P* < .001), resting dendritic cells (*P* = .013), resting mast cells (*P* = .001) and neutrophils (*P* < .001) (Figure 2B). Additionally, we observed that the proportion of Treg cells (*P* = .025) and M0 macrophages (*P* = .004) was higher in PCa tissues compared to normal tissues (Figure 2B).

**Figure 3:**
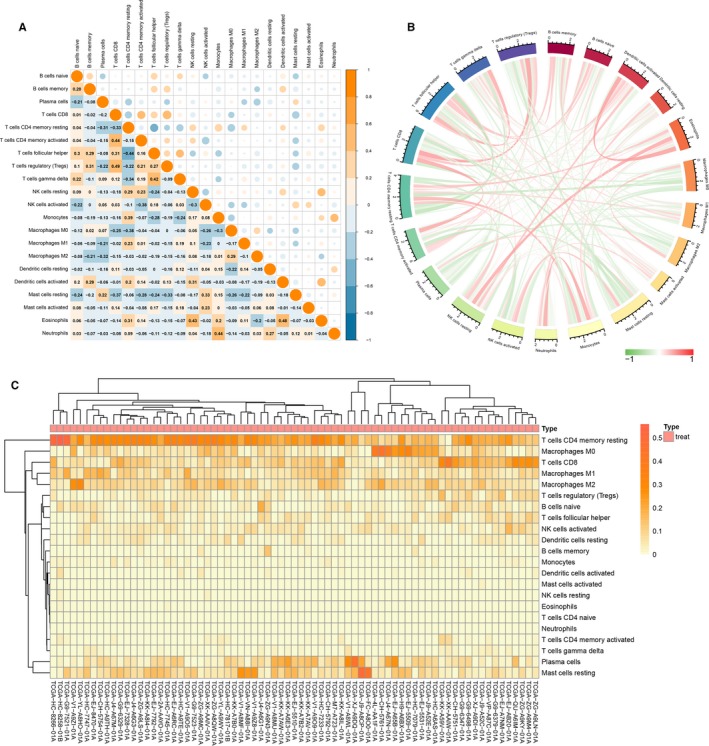
The landscape of immune infiltration in prostate cancer. A and B, Correlation matrix of all 21 immune cell densities in the TCGA cohort. C, Heat map of the 21 immune cell proportions. The horizontal axis displays the grouping data of samples divided into two major categories

To determine the underlying relationships of different immune types, we evaluated the correlation between TIICs among PCa tissues. Figure 3A shows the R value between every two types of immune cells with digits and bubbles, the orange bubbles show a positive correlation, while the blue bubbles show a negative correlation, what is more is the size of each bubble indicated the R value. The two most relevant TIICs are Treg cells and CD8^+^ T cells, with an R value of 0.49. Additionally, CD8^+^ T cells were positively associated with activated CD4^+^ memory T cells (R = 0.44), and neutrophils were linked with monocytes (R = 0.44). In contrast, the existence of resting CD4^+^ memory T cells was negatively associated with follicular helper T cells (R = −0.44), activated NK cells, activated CD4^+^ memory T cells (R = −0.38), resting CD4^+^ memory T cells and M0 macrophages (R = −0.38). Figure 3B also shows the correlations between immune cells, the line weight represents the degree of correlation. Besides, as shown in Figure 3C, PCa samples were split into two discrete groups using unsupervised hierarchical clusters based on the above identified cell subpopulations. These findings suggest that aberrant and heterogeneous immune infiltration within PCa could have important clinical significance as a tightly regulated process.

### 3.2 Immune infiltration with clinical characteristics and PCa recurrence

**Figure 4:**
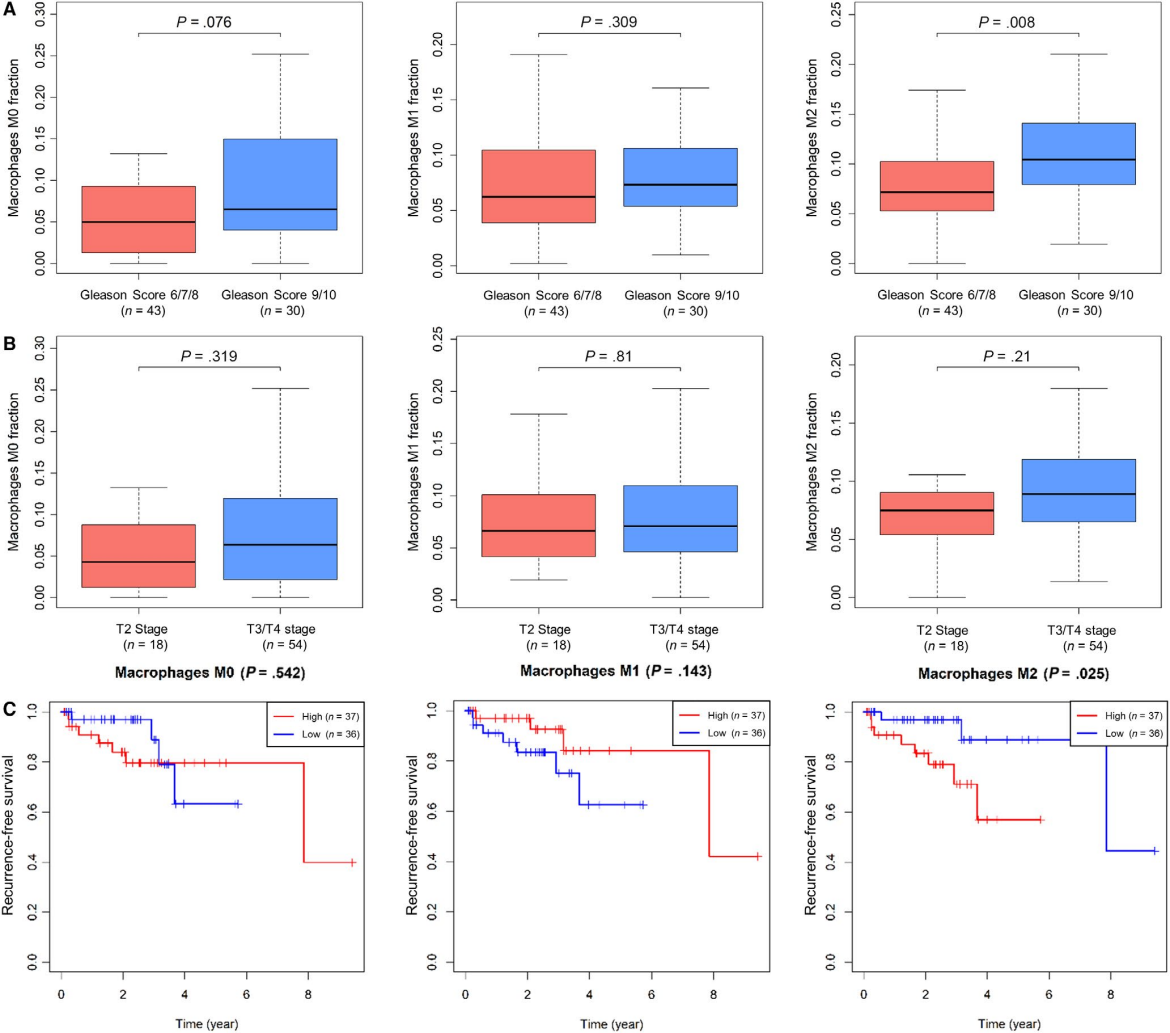
Immune infiltration in different clinical stages and recurrence‐free survival (RFS). Macrophage distribution in different Gleason scores (A) and pathology T stage (B); K‐M curves show the different RFS in the high‐ and low‐density of three types of macrophage. *P‐values* are from log‐rank tests. [Correction added on 5 August 2019, after first online publication: In Figure 4C, the legend (Red and Blue line) that represent “High risk” and “Low risk” was interchanged. This has been corrected in this version.]

We downloaded the clinical characteristics of PCa patients from the TCGA database and extracted the main clinical items, including patient ID, age, Gleason score, pathology T stage, pathology N stage, tumor histological type, the status of tumor recurrence and recurrence‐free time. All the clinical features were listed in Table 1. For macrophage, we found that the three types of macrophages have a higher proportion in the advanced PCa, especially for M0 macrophage in 9 + 10 Gleason score group (*P* = .076), M2 macrophage in Gleason 9 + 10 group (*P* = .008) (Figure 4A). Additionally, an exciting result among plasma cells, Treg cells, and resting mast cells was revealed in the Gleason subgroup, T stage subgroup and N stage subgroup. Plasma cells displayed a downregulated status in PCa tissues in patients with severe tumor status, including the 9 + 10 Gleason score group (*P* = .042), T3 + T4 stage (*P* = .36). Treg cells were high in tumor tissues among severe stages, including the 9 + 10 Gleason score group (*P* = .009), T3 + T4 stage (*P* = .044), while resting mast cells showed a lower density among the 9 + 10 Gleason score group (*P* = .004), T3 + T4 stage (*P* = .027) (Figure S1).

**Table 1:** Clinical characteristics of prostate cancer patients from TCGA database

	Recurrence (n = 11)	No recurrence (n = 62)	Total
Age (years)	61.73 ± 5.20	61.37 ± 6.95	61.42 ± 6.68
Gleason score, n (%)			
6 + 7+8	6 (8.22)	38 (52.05)	44 (60.27)
9 + 10	5 (6.85)	24 (32.88)	29 (39.73)
Pathology T stage^a^, n (%)			
T2	0	19 (26.03)	18 (24.66)
T3 + T4	11 (15.07)	43 (58.90)	54 (73.97)
Pathology N stage^b^, n (%)			
N0	6 (8.22)	38 (52.05)	44 (60.27)
N1	5 (6.85)	16 (21.92)	21 (28.77)
Histological type, n (%)			
Adenocarcinoma Acinar	11 (15.07)	60 (82.19)	71 (97.26)
Others	0	2 (2.74)	2 (2.74)

a T stage of 1 patient was not available.

b N stage of 8 patients was not available.

PCa is a mild cancer with a long survival time around the world. However, biochemical recurrence and hormone‐refractory PCa are still urgent problems. We analyzed the correlation between immune infiltrations with the RFS time among the enrolled patients. We found that a higher proportion of M2 macrophages in PCa is associated with poor prognosis (mean RFS = 819.11 days), while a lower proportion indicated a more prolonged RFS (mean RFS = 992.65 days, *P* = .025) (Figure 4C). There were no differences in RFS between the low proportion and high proportion of the other TIICs.

### 3.3 Establishment of PCa recurrence prediction nomogram

**Figure 5:**
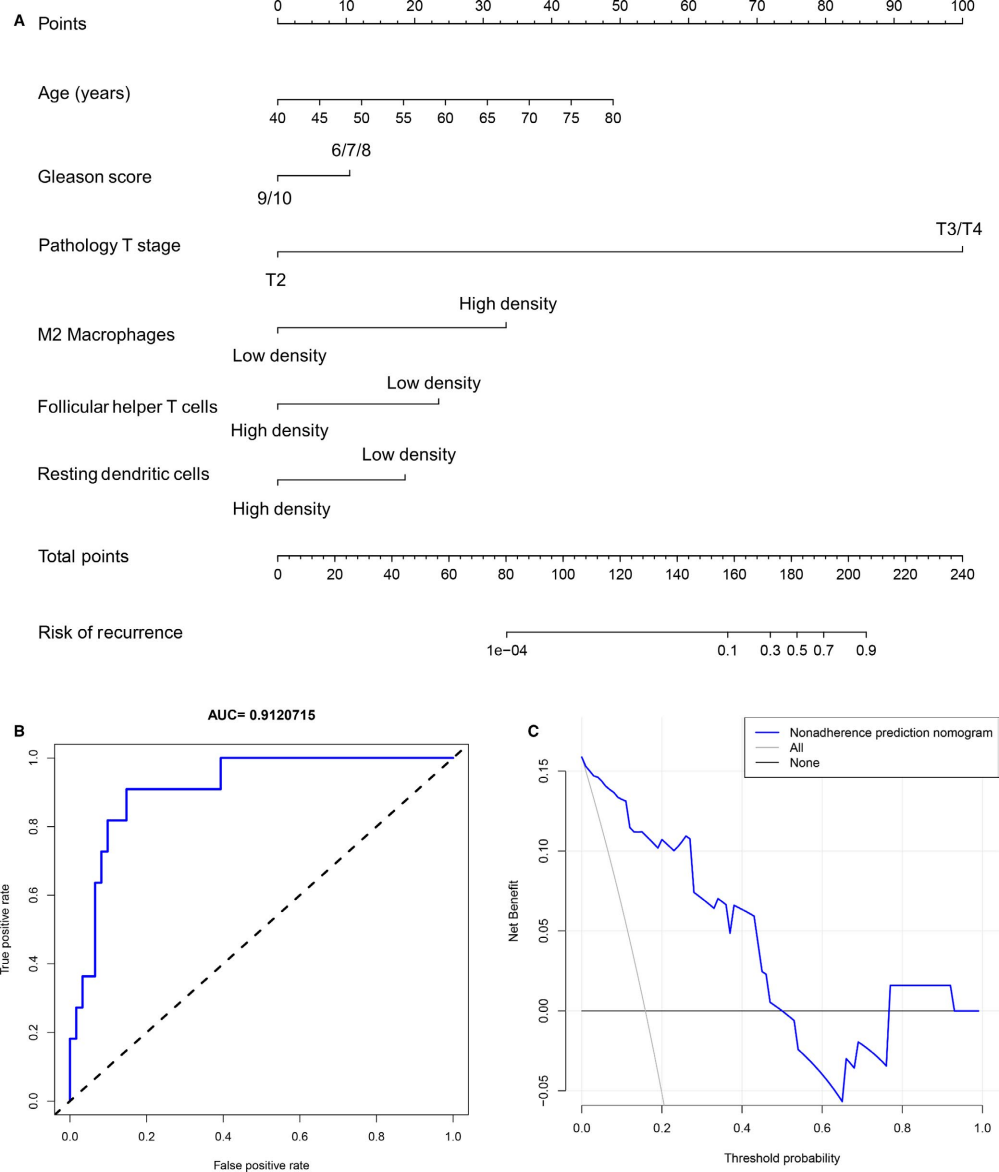
Established prostate cancer (PCa) recurrence prediction nomogram. A, Developed nomogram to predict the recurrence risk of PCa patients based on clinical parameters and proportions of tumor‐infiltrating immune cells. B, ROC curve performed to assess the performance of the PCa recurrence predictive nomogram. C, Decision curve analysis for the PCa recurrence predictive nomogram

To establish a convenient method to predict the recurrence risk of PCa, we designed a nomogram based on clinical information and density of TIICs. First, the subtypes of TIICs were selected by LASSO regression analysis, including M2 macrophages, follicular helper T cells and resting dendritic cells (Figure S2). In addition, we established the recurrence prediction nomogram based on the infiltration density of three TIICs, age, Gleason score and pathology T/N stages. TIICs were divided into high‐ and low‐risk groups based on the cutoff value of their effect on PCa recurrence based on a ROC curve. As shown in Figure 5A, the top "points" bar is the scale to estimate the risk score of each parameter, and the bottom "total points" bar corresponds to the risk of PCa recurrence. For each patient, we obtain the 7 points from the enrolled items, and the total points may indicate the risk of PCa recurrence. To assess the accuracy of the nomogram, ROC curve and the area under the ROC curve (AUC) were performed, and the AUC value is 0.912 in the PCa patient prediction cohort (Figure 5B). Furthermore, we performed a decision curve analysis to evaluate the clinical utility of the nomogram, and the result is shown in Figure 5C. The DCA curve demonstrated that the nomogram had benefits more than the treat‐all‐patients or treat‐none strategy as the threshold probability for a patient ranged from 0 to 0.5. All the results reflect that the nomogram could precisely and steadily judge the risk of PCa recurrence.

### 3.4 Correlation between M2 macrophage infiltration and androgen receptor

AR plays pivotal roles in the biochemical recurrence and drug resistance of PCa. As a high proportion of M2 macrophage infiltration is positively associated with the shorter RFS, we evaluated the relationship of AR and M2 macrophages infiltration, as well as AR‐related genes. The five main AR‐related proteins were identified with STRING, an online website that illustrates the correlation between proteins. All of the information was based on curated databases, experimentally determined or text mining. Finally, we recorded KLK3, KDM1A, NCOA2, NCOA4, and FOXA1 (Figure S3). Furthermore, we evaluated the expression of AR and the five genes mentioned above in TIMER, an online interactive immune estimation resource, to identify the underlying links between the genes and M2 macrophages. The tumor purity index is the ratio of tumor cells in the tumor tissues. A lower purity indicates a mixture of other cells, such as TIICs. To assess the correlation between macrophages and the genes, we adjusted and excluded the impact of tumor purity. As shown in Figure S4, the X‐axis indicated the infiltration level of macrophages, and the Y‐axis represented the expression level of the genes. The proportion of macrophages was definite for AR (R = 0.379, *P* < .001), after adjusting for tumor purity, and similar results were illustrated for NCOA2 (R = 0.392, *P* < .001) and NCOA4 (R = 0.165, *P* < .001). In contrast, the expression of FOXA1 (R = −0.210, *P* < .001), KDM1A (R = −0.256, *P* < .001) and KLK3 (R = −0.247, *P* < .001) were negatively associated with the distribution of macrophages in PCa tissues.

**Figure 6:**
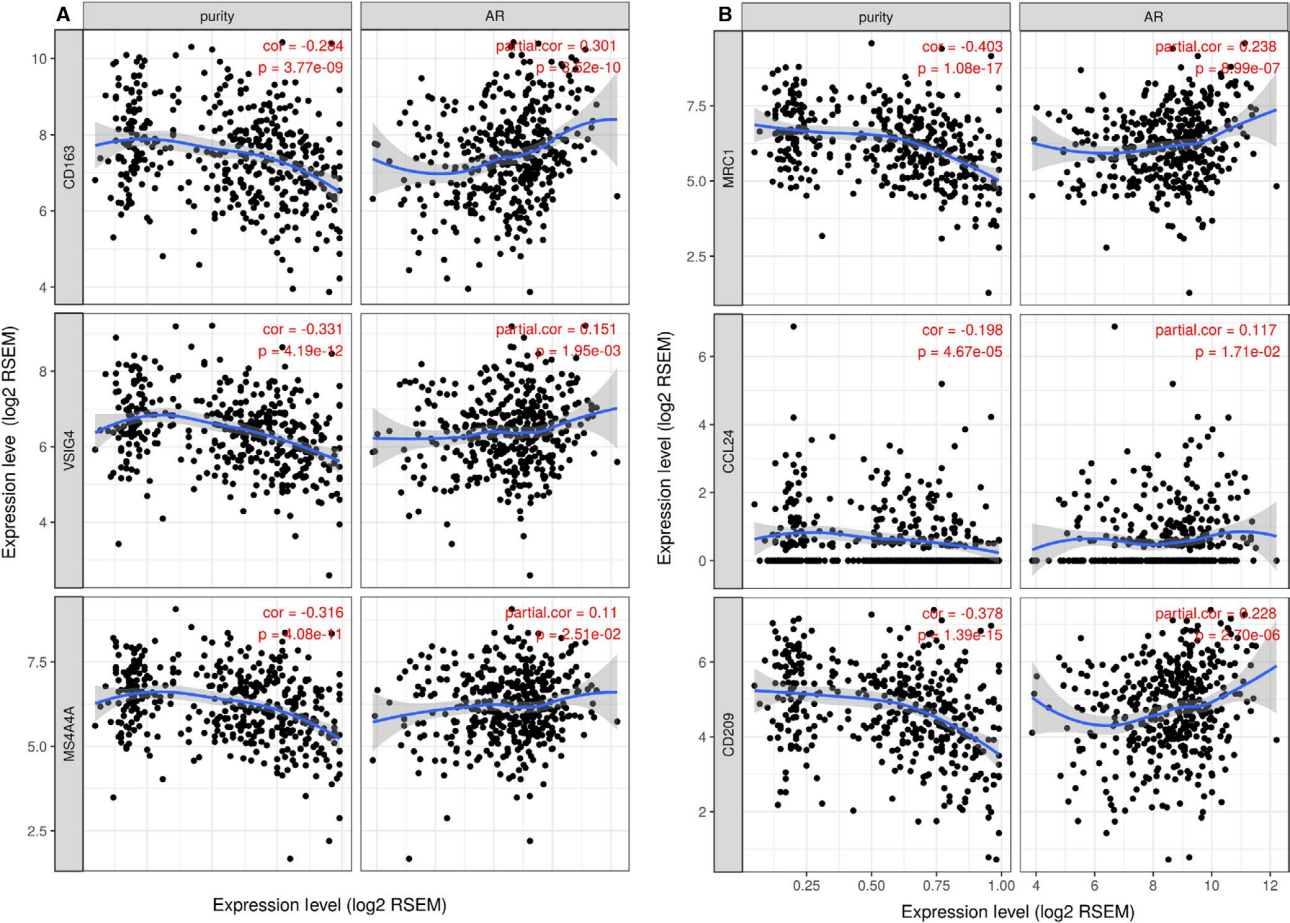
AR and M2 macrophage markers. TIMER was conducted to assess the co‐expression of AR and M2 macrophage markers, such as CD163 (A), MRC1 (B), VSIG4 (C), CCL24 (D), NS4A4A (E), and CD209 (F)

Additionally, we compared the correlation of M2 macrophage gene markers and AR, as well as tumor purity. The maker gene of M2 macrophage was selected from the prior publications.^25^, ^26^ The tumor purity was negatively linked with the expression of CD163, VSIG4, MS4A4A, MRC1, CCL24, and CD209, indicating that the result is reliable, because the higher tumor purity represented a lower proportion of TIICs, including M2 macrophages. Alternatively, the result showed that AR expression is positively related with the expression of CD163 (R = 0.301, *P* < .001), VSIG4 (R = 0.151, *P* = .002), MS4A4A (R = 0.110, *P* = .025), MRC1 (R = 238, *P* < .001), CCL24 (R = 0.117, *P* = .017), and CD209 (R = 0.228, *P* < .001) (Figure 6). These results show the potential correlation between M2 macrophages and AR, as well as AR‐related genes, whether there is the regulate function and how the mechanism is need to be investigated based on the experiment in the future.

## 4 DISCUSSION

Immune infiltration plays a critical role in tumor progression. Immune evasion is now regarded as a cancer hotspot. One of the critical mechanisms is the programmed cell death protein (PD‐1) receptor on cytotoxic T lymphocytes (CTLs) and its ligand (PD‐L1) on cancer cells or host TIICs. Immunotherapies targeting PD1/PD‐L1, which can reverse immune tolerance and yielded remarkable clinical responses, have been widely studied in tumors.^27^, ^28^, ^29^, ^30^ Zheng et al^31^ indicated that the balance of Th17 cells and Treg cells was broken in patients who underwent gastric cancer resection, increasing the function of the PD‐1/PD‐L1 axis. Scimeca et al^32^ demonstrated a decrease of both PD‐1 positive lymphocytes and tumor‐infiltrating macrophages, primarily the M2 subtype, as an inflammatory infiltration of PD‐L1 positive prostate lesions. Regarding other types of immune response, Sage et al^33^ reported that after receiving radiotherapy treatment, lymphocyte counts decreased, especially the density of B cells, while the T cell and NK cell percentages were unchanged. Eckert et al^34^ demonstrated that radiotherapy could downregulate the counts of leukocytes, lymphocytes, B cells, CD8^+^ cells, and naïve CD4^+^ T cells, while Treg cells and NK cells increased. Regarding macrophages, Shimura et al^35^ determined that a high density of macrophages in both tumor stroma and cancer areas was negatively associated with advanced stages of PCa comparing TNM stage, extra prostatic extension, seminal vesicle invasion, lymph node metastasis, and surgical margin. In contrast, recent studies reported that tumor‐associated macrophage infiltration is a predictive signature of patients who underwent hormonal therapy and that the tumor‐associated macrophage proportion is higher in patients with higher serum PSA, higher Gleason score, more severe T stage, and PSA recurrence.^36^

In previous studies, most researchers detected the density of TIIC proportion based on fewer specific markers. However, several papers demonstrated the overlap of molecular markers for TIICs.^37^, ^38^, ^39^ What is more are the positive counts of TIICs determined by the individual, and the IHC method limits the types of TIICs that can be detected on one tissue section. In the current research, the density of 22 main TIICs in PCa and control tissues was evaluated based on the deconvolution algorithm of CIBERSORT. Finally, the proportion of 73 PCa tissues and 11 controls were confirmed by appropriate standards. Twenty‐one types of TIICs were detected in patients, while naïve CD4^+^ T cells did not appear in any patient or control. We found that activated CD4^+^ T cells and M0 macrophages are found at high levels in tumor tissues, while neutrophil and monocyte levels are lower, and these results are consistent with previous studies.

In the second part of the study, we illustrated the TIICs in PCa tissues and their internal correlation. The features of patients are divided into two parts with unsupervised hierarchical clustering, meaning that the different proportions of TIICs in PCa are associated with the clinical results. We also determined that CD8^+^ T cells are positively associated with activated CD4^+^ memory T cells, while resting CD4^+^ memory T cells are negatively associated with follicular helper T cells. Connie et al^40^ previously illustrated that Th1 cells, a type of CD4^+^ memory T cells, are required to enhance the primary activated process of CD8^+^ T cells in immune responses, while Edith et al^41^ reported that CD4^+^ T cells are required for secondary expansion and memory of CD8^+^ T cells. Follicular helper T cells are distinguished from CD4^+^ T cells, which function in the formation of germinal centres and promote the proliferation and differentiation of B cells. Hale et al^42^ found that there are distinct memory CD4^+^ T cell populations that commit to follicular helper T cell lineages, and CD4^+^ memory T cells can recall their previous lineage of effectors provided by follicular T cells.

Subsequently, the analysis of TIICs and clinical features was performed, and we found that the proportion of plasma cells and mast cells was downregulated in severe PCa, compared to mild PCa, while the density of Treg cells was upregulated. Lohr et al^43^ reported that mature plasma cells detected in the immune‐infiltrated tumor tissues were associated with a prolonged survival time of small cell lung cancer. For mast cells, Anna et al^44^ determined that mast cells were found at high levels in PCa tissues, and they can promote the process of PCa through the angiogenic factor FGF‐2, the results of which were not consistent with the current 547 specific gene‐based results. Flammiger et al^45^ marked Treg cells in PCa tissues using FOXP3 immunohistochemistry, and the results showed that PSA recurrence‐free survival was decreased in patients with a higher density of Treg cells, and a high level of FOXP3^+^ Treg cells was associated with advanced PCa tumor stage. Erlandsson et al^46^ found that M2 macrophages and Treg cells are independent lethal factors for PCa and that these two types of TIICs could promote an immunosuppressive environment.

After assessing the association between TIICs and RFS of PCa patients, M2 macrophages were singled out. Patients whose M2 macrophage density is higher had a shorter RFS time. Although the K‐M curve of M0 and M1 macrophages showed a similar trend, no statistical significance was found. Feng et al^47^ also reported the landscape of PCa patients based on several GEO databases, which is consistent to the current study. We found that M2 macrophage is a risk factor to PCa patients, Feng et al also reported that the higher ratio of M1 + M2/M0 indicated a poor prognosis. In addition, we built a nomogram to predict the risk of recurrence based on the immune infiltration and clinical data. Finally, M2 macrophages, follicular helper T cells and resting dendritic cells were identified, while age, Gleason score and pathology T/N stages were also used to assess the recurrence. ROC and DCA were performed to verify that the novel nomogram is discriminatory and has utility. Moreover, we evaluated whether the key genes involved in PCa recurrence were related to the proportion of macrophages. The result showed that AR, NCOA2, and NCOA4 were positively correlated with the proportion of macrophages, while FOXA1, KDM1A, and KLK3 displayed a negative association. The expression of M2 macrophage‐specific protein markers increased with the upregulation of AR after adjusting for tumor purity. The function of macrophages in PCa has been widely reported. Philippe et al^22^ demonstrated that the high macrophage density was linked to increasing biochemical recurrence risk. Gollapudi et al^48^ illustrated a controversial point. They found that tumor‐associated macrophage infiltration is associated with tumors compared with benign prostate tissues, as well as a higher Gleason score, but no evidence linked the positive expression of macrophages and biochemical recurrence after radical prostatectomy. Fang et al^49^ reported an interesting phenomenon where the co‐culture of prostate epithelial cells and macrophages could induce tumorigenesis via AR‐mediated CCL4‐STAT3 signaling pathways. In a study conducted by Izumi et al, metastasis promotion enhanced through macrophage recruitment by AR knockdown was also revealed. The potential mechanisms might be CCL2/CCR2‐induced STAT3 activation.

In summary, using the deconvolution algorithm of CIBERSORT, we first obtained the distribution of 22 TIICs in PCa patients based on the expression of 547 core genes. The proportion of immune infiltration was significantly different between PCa and control tissues. Plasma cells, Treg cells, and mast cells are involved in the progression of PCa, while M2 macrophages could predict the prognosis of PCa patients. Meanwhile, based on the LASSO regression analysis, we established a novel nomogram to assess the recurrence risk of PCa based on immune cell proportions and clinical features, of which need to be verified by a large size cohort in the feature.

## CONFLICT OF INTEREST

All authors declared no competing interests.

## AUTHOR CONTRIBUTIONS

JLM, MZ, and CZL conceived and designed the study. MZ, YL, and SF performed the data extraction. JLM, MZ, and SYG analyzed the data. YL, SF, and ZYH contributed materials and analysis tools. JLM, JZ, JZ, and CZL wrote the manuscript. All authors have read and approved the final version of the manuscript and agreed with the order of presentation of the authors.
