# Supplementary material for: The establishment of immune infiltration based novel recurrence predicting nomogram in prostate cancer
Source: Cancer Med. 2019 Jul 29;8(11):5202–13. doi: 10.1002/cam4.2433 (PMC6718526; doi:10.1002/cam4.2433)
Supplement: Supplementary file 1 [file CAM4-8-5202-s001.pdf]

Supplementary data

The establishment of Immune Infiltration Based Novel Recurrence Predicting Nomogram in Prostate Cancer

Jialin Meng[1]; Yi Liu[1], Shiyang Guan[2]; Song Fan[1]; Zongyao Hao[1], Jun Zhou[1]; Meng Zhang[1*], Chaozhao Liang[1*]

[1] Department of Urology, The First Affiliated Hospital of Anhui Medical University; Institute of Urology, Anhui Medical University; Anhui Province Key Laboratory of Genitourinary Diseases, Anhui Medical University, 218th Jixi Road, Hefei, Anhui, China.

[2] Department of Epidemiology and Biostatistics, School of Public Health, Anhui Medical University, 81th Meish an Road, Hefei, Anhui, China

[*]**Corresponding author:**

Chaozhao Liang, MD, PhD, No.218, Jixi Road, Hefei 230022, Anhui, China; Tel.: +86 55162923440; Email address: liang_chaozhao@ahmu.edu.cn

Meng Zhang, PhD, No.218, Jixi Road, Hefei 230022, Anhui, China; Tel.: +86 55162922234; Email address: zhangmeng1930@163.com

Running title: Immune Infiltration and Recurrence Nomogram to Prostate Cancer

Table S1. The proportions of TIICs in PCa and control tissues.

id	B cells naive	B cells memory	Plasma cells	T cells CD8	T cells CD4 naive
TCGA-EJ-7794-11A	0.043554513	0	0.071499736	0.06602704	0
TCGA-EJ-7783-11A	0.025304794	0	0.046455094	0	0
TCGA-EJ-7330-11A	0.057014574	0	0.031017155	0.142829171	0
TCGA-EJ-7331-11A	0.03812147	0	0.043943426	0.063424869	0
TCGA-EJ-7782-11A	0.096365609	0	0.085431701	0.074470666	0
TCGA-EJ-7315-11A	0.06617335	0	0.0327095	0.099996038	0
TCGA-EJ-7792-11A	0.058113244	0	0.09451064	0.046853441	0
TCGA-EJ-7781-11A	0.006165361	0	0.043394266	0.04490343	0
TCGA-EJ-7328-11A	0.065799501	0	0.030923741	0.071745124	0
TCGA-EJ-7125-11A	0.008791734	0	0.095398459	0.079299776	0
TCGA-HC-7752-11A	0.025212988	0	0.029094363	0.112007916	0
TCGA-V1-A8WL-01A	0.076421256	0	0.290356623	0.030803701	0
TCGA-HC-7745-01A	0.014672189	0	0.068818261	0.069524369	0
TCGA-V1-A9ZI-01A	0.072973233	0	0.023961299	0.074704973	0
TCGA-VN-A88I-01A	0.042691914	0.005058349	0.001076858	0.149355982	0
TCGA-G9-6379-01A	0.014903193	0	0.053195005	0.143969144	0
TCGA-G9-6347-01A	0.055102753	0	0.088294528	0.205816718	0
TCGA-HC-8266-01A	0.010600233	0	0.009835716	0.186216094	0
TCGA-HC-7233-01A	0.020576479	0	0.036915436	0.15332629	0
TCGA-KK-A7B2-01A	0.007487706	0	0.132914618	0.095067212	0
TCGA-VP-A87C-01A	0.108606064	0	0.008314859	0.15768631	0
TCGA-J4-A6G1-01A	0.196070287	0.029620781	0.086943697	0.073178393	0
TCGA-KK-A7AV-01A	0.011266975	0.03235991	0.146296436	0.121637907	0
TCGA-J9-A52E-01A	0.010412489	0	0.089528527	0.10991699	0
TCGA-EJ-7328-01A	0.016062487	0	0.058871159	0.131489672	0
TCGA-EJ-7792-01A	0.088959911	0	0.085132589	0.059353195	0
TCGA-ZG-A8QW-01A	0.032678804	0	0.012799595	0.106416007	0
TCGA-EJ-8470-01A	0.072161071	0	0.041873719	0.044658185	0
TCGA-V1-A8MU-01A	0.005828578	0	0.179062398	0.112896761	0
TCGA-KK-A6E3-01A	0	0	0.225570968	0.061749232	0
TCGA-4L-AA1F-01A	0.035539606	0	0.019470279	0.038202682	0
TCGA-EJ-5531-01A	0.046631549	0	0.121081411	0.108058577	0
TCGA-ZG-A9LS-01A	0.035214564	0.030767495	0.014066603	0.132828674	0
TCGA-CH-5754-01A	0.038145721	0	0.091494679	0.012580513	0
TCGA-J9-A8CP-01A	0.025284309	0	0.214330089	0.035164806	0
TCGA-ZG-A9M4-01A	0.019933048	0	0.061614909	0.275826209	0
TCGA-M7-A723-01A	0.046527925	0	0.13019542	0.156990536	0
TCGA-KK-A8I4-01A	0.040929125	0	0.016002778	0.119666048	0
TCGA-EJ-A7NM-01A	0.050188259	0.031233219	0.013845242	0.15535747	0
TCGA-V1-A9O9-01A	0.016255667	0	0.176385803	0.14271189	0
TCGA-YL-A9WY-01A	0.006281414	0	0.02487321	0.114819568	0
TCGA-KK-A59V-01A	0.026846667	0.002629454	0.01565909	0.305709944	0
TCGA-V1-A8MF-01A	0.040417237	0	0.067049236	0.129172632	0
TCGA-EJ-AB20-01A	0.037099922	0	0.012389203	0.202328113	0
TCGA-ZG-A9N3-01A	0.09603882	0.13706982	0.041519755	0.098134645	0
TCGA-EJ-A6RC-01A	0.09734116	0	0.12304731	0.043773846	0
TCGA-XJ-A9DQ-01A	0	0	0.447206774	0.05155602	0
TCGA-YL-A8HO-01A	0.067743961	0	0	0.082353457	0
TCGA-ZG-A9MC-01A	0.045837745	0.047148511	0	0.079687066	0
TCGA-XK-AAIV-01A	0.032039925	0.010465577	0.022443795	0.031608302	0
TCGA-ZG-A9KY-01A	0.03736968	0	0.005742518	0.266405652	0
TCGA-XK-AAIW-01A	0.057007896	0	0.048469167	0.405286847	0
TCGA-QU-A6IM-01A	0	0	0.148982696	0.295952758	0

id	T cells CD4 memory resting	T cells CD4 memory activated	T cells follicular helper	T cells regulatory (Tregs)	T cells gamma delta	NK cells resting
TCGA-EJ-7794-11A	0.30585237	0	0.00522738	0.03117901	0	0.00559192
TCGA-EJ-7783-11A	0.41029242	0	0	0.00038389	0	0
TCGA-EJ-7330-11A	0.2126391	0	0.02898955	0	0.01405321	0
TCGA-EJ-7331-11A	0.30542888	0	0.01339294	0.01299583	0	0
TCGA-EJ-7782-11A	0.23822623	0.01865646	0.04849047	0.01007675	0.02647978	0
TCGA-EJ-7315-11A	0.32518541	0	0.04828017	0.0173636	0.00089027	0
TCGA-EJ-7792-11A	0.33266231	0	0.03843488	0	0	0
TCGA-EJ-7781-11A	0.38670019	0	0.03542102	0.00394536	0	0
TCGA-EJ-7328-11A	0.4078316	0	0.00070122	0	0	0
TCGA-EJ-7125-11A	0.12384193	0	0.04382102	0	0.06207795	0
TCGA-HC-7752-11A	0.28729708	0	0.02172033	0.06155785	0	0
TCGA-V1-A8WL-01A	0.16418528	0.01015274	0.023768	0.0074206	0.06081298	0
TCGA-HC-7745-01A	0.32488413	0	0.01680541	0	0	0
TCGA-V1-A9ZI-01A	0.20532207	0.01064347	0	0.02272193	0	0.01008023
TCGA-VN-A88I-01A	0.30705592	0	0	0.0368714	0	0
TCGA-G9-6379-01A	0.18670913	0	0.06351236	0.08213269	0.03175773	0
TCGA-G9-6347-01A	0.22993768	0	0.01635756	0.06402733	0	0
TCGA-HC-8266-01A	0.51243625	0	0.00034963	0.05241083	0	0
TCGA-HC-7233-01A	0.30270512	0.02029418	0.0261684	0.00980564	0	0
TCGA-KK-A7B2-01A	0.21183337	0	0.03528481	0.01233043	0.01596686	0
TCGA-VP-A87C-01A	0.18246982	0.02398077	0.09553963	0.07300553	0.03676161	0
TCGA-J4-A6G1-01A	0.24449481	0.00107936	0.09124597	0.03545139	0.02785592	0
TCGA-KK-A7AV-01A	0.21779157	0.01381765	0.05675799	0.04221058	0	0
TCGA-J9-A52E-01A	0.17652941	0	0.03976167	0.07262397	0	0
TCGA-EJ-7328-01A	0.32225698	0	0	0.01351339	0	0
TCGA-EJ-7792-01A	0.27032925	0	0.04029136	0.02296971	0	0
TCGA-ZG-A8QW-01A	0.35908024	0.01796061	0.03359613	0.00085254	0	0
TCGA-EJ-8470-01A	0.2874854	0	0.09242347	0	0.04111246	0
TCGA-V1-A8MU-01A	0.20816693	0	0	0.0150474	0.0326278	0
TCGA-KK-A6E3-01A	0.22860136	0	0.08385605	0	0.02074334	0
TCGA-4L-AA1F-01A	0.15801558	0	0.02470584	0.01367156	0.00770941	0
TCGA-EJ-5531-01A	0.14922192	0	0.01031505	0.02413612	0	0
TCGA-ZG-A9LS-01A	0.33154194	0.00235601	0.01956629	0.05776052	0	0
TCGA-CH-5754-01A	0.31350989	0	0.04337377	0	0.01492778	0
TCGA-J9-A8CP-01A	0.08694008	0	0.00857673	0.01036663	0	0
TCGA-ZG-A9M4-01A	0.11603984	0	0.11202461	0.08742611	0.01436693	0
TCGA-M7-A723-01A	0.20367405	0	0.00367787	0.0019182	0	0
TCGA-KK-A8I4-01A	0.3213662	0	0	0.04342799	0	0
TCGA-EJ-A7NM-01A	0.22404742	0	0.03781709	0.1378469	0	0
TCGA-V1-A9O9-01A	0.36006233	0	0	0.0141108	0	0.00616949
TCGA-YL-A9WY-01A	0.30217265	0.03805699	0.0018199	0	0.02297035	0
TCGA-KK-A59V-01A	0.0418261	0.0924908	0.08432878	0.15786301	0.02430997	0
TCGA-V1-A8MF-01A	0.19830438	0.00386399	0.0160622	0.00808556	0	0
TCGA-EJ-AB20-01A	0.25570019	0	0.10378843	0	0.02416073	0
TCGA-ZG-A9N3-01A	0.23670888	0	0.07432888	0.07748217	0	0
TCGA-EJ-A6RC-01A	0.34504208	0	0.03729778	0.02638989	0	0
TCGA-XJ-A9DQ-01A	0.21022074	0	0.00640681	0	0.00509546	0
TCGA-YL-A8HO-01A	0.29837717	0	0.02568131	0	0.00999429	0
TCGA-ZG-A9MC-01A	0.30940549	0	0.04903906	0.05498651	0	0
TCGA-XK-AAIV-01A	0.32626257	0.03162244	0	0.04052582	0	0.0294924
TCGA-ZG-A9KY-01A	0.16389186	0.00146851	0.05580402	0.08494525	0	0
TCGA-XK-AAIW-01A	0	0.07995535	0.10244816	0.01627595	0.03853712	0
TCGA-QU-A6IM-01A	0.14628247	0	0.04463149	0.08237401	0	0

id	NK cells activated	Monocytes	Macrophages M0	Macrophages M1	Macrophages M2	Dendritic cells resting
TCGA-EJ-7794-11A	0.00584687	0.05451566	0.07158877	0.07850739	0.08501824	0.03409897
TCGA-EJ-7783-11A	0.06148568	0.04846098	0	0.00571876	0.11911677	0
TCGA-EJ-7330-11A	0.01696504	0.04895799	0.01113161	0.04774431	0.10569691	0.09116387
TCGA-EJ-7331-11A	0.01634337	0.02606109	0	0.07543558	0.10990194	0.06374306
TCGA-EJ-7782-11A	0.02309924	0.00580583	0.03707897	0.09402223	0.05596361	0.09119774
TCGA-EJ-7315-11A	0.05398294	0.00636474	0.04265599	0.06567113	0.05236837	0.0970888
TCGA-EJ-7792-11A	0.02639106	0.05170194	0.00056709	0.06225465	0.10464261	0.04814098
TCGA-EJ-7781-11A	0.08537632	0.02754471	0.05468384	0.04326472	0.11308957	0.00761081
TCGA-EJ-7328-11A	0.03501344	0.01911269	0	0.13111442	0.06625207	0.0319245
TCGA-EJ-7125-11A	0.07595569	0.01772449	0.03059494	0.1449376	0.05470904	0.06448999
TCGA-HC-7752-11A	0.04874091	0.01161614	0.01800358	0.09894713	0.09956773	0.03314563
TCGA-V1-A8WL-01A	0.02176775	0.00298074	0.07479317	0.07670248	0.020601	0.00626142
TCGA-HC-7745-01A	0.07197785	0.0368483	0.06477867	0.13351519	0.01391274	0.0421901
TCGA-V1-A9ZI-01A	0	0	0.19002243	0.04625971	0.27633847	0.01162363
TCGA-VN-A88I-01A	0.08919476	0.0042062	0.00481502	0.00217774	0.11379571	0.00251388
TCGA-G9-6379-01A	0.0526334	0.00022106	0.0662324	0.05899905	0.10005339	0.03754207
TCGA-G9-6347-01A	0.04909773	0.01204453	0.04492778	0.02406833	0.10589337	0
TCGA-HC-8266-01A	0	0.01687921	0.01372454	0.12997591	0.04892363	0.01636344
TCGA-HC-7233-01A	0.01417438	0.0118038	0.0313092	0.12538006	0.17395751	0.00423343
TCGA-KK-A7B2-01A	0.08578726	0.01611217	0.05405189	0.11091407	0.07211796	0.05319868
TCGA-VP-A87C-01A	0.00637811	0	0.0701956	0.07879149	0.05945463	0.05390582
TCGA-J4-A6G1-01A	0.0120041	0	0.10498163	0.01879558	0.03210777	0
TCGA-KK-A7AV-01A	0.02235179	0	0.0311935	0.10077255	0.06965853	0.01680409
TCGA-J9-A52E-01A	0.0034921	0	0.20709338	0.06457045	0.16360889	0.00343142
TCGA-EJ-7328-01A	0.04116206	0.0377692	0.06360506	0.1125351	0.05607435	0.04150693
TCGA-EJ-7792-01A	0.04957089	0.01771071	0.04533896	0.03225447	0.10140912	0.0191815
TCGA-ZG-A8QW-01A	0.04782314	0.00532008	0.10280996	0.06982065	0.11585596	0.04729577
TCGA-EJ-8470-01A	0.01522297	0	0.13217894	0.16236163	0.08057792	0.00883974
TCGA-V1-A8MU-01A	0.04658471	0.00448507	0.09805088	0.05812326	0.05113723	0.02225004
TCGA-KK-A6E3-01A	0.05052774	0.00093435	0.04109246	0.04956064	0.05418998	0.01935179
TCGA-4L-AA1F-01A	0.02417068	0	0.39374551	0.09240679	0.15096651	0
TCGA-EJ-5531-01A	0.01490426	0.02412448	0.21709043	0.01433117	0.0698304	0.08170839
TCGA-ZG-A9LS-01A	0.05780327	0.00632824	0.01947886	0.05378121	0.08454133	0.07222845
TCGA-CH-5754-01A	0.02426877	0.01921453	0.0579318	0.20259914	0.09238585	0.02668124
TCGA-J9-A8CP-01A	0.05690145	0	0	0.02389996	0.0327303	0.00318101
TCGA-ZG-A9M4-01A	0.05687515	0.00185616	0.06517175	0.07600597	0.05357618	0.04301743
TCGA-M7-A723-01A	0.0243959	0.00307459	0	0.17006825	0.13607936	0.03871974
TCGA-KK-A8I4-01A	0.05482636	0.01648651	0.04978088	0.08462245	0.07329613	0.04202356
TCGA-EJ-A7NM-01A	0.04744642	0.01270147	0.06238153	0.04262339	0.07375443	0.05316885
TCGA-V1-A9O9-01A	0	0	0.06541419	0.13844779	0.04686541	0.00782932
TCGA-YL-A9WY-01A	0.00582846	0	0.1498102	0.07866678	0.18016375	0.02521349
TCGA-KK-A59V-01A	0.00385706	0	0.09090428	0.08122229	0.04641693	0.00104834
TCGA-V1-A8MF-01A	0.02549582	0.00383088	0.01547276	0.06595602	0.07569227	0.09298318
TCGA-EJ-AB20-01A	0.1720146	0.02076211	0	0.02949325	0.06409636	0.01927291
TCGA-ZG-A9N3-01A	0.05424858	0	0.04794855	0.05125861	0.01935082	0.01882043
TCGA-EJ-A6RC-01A	0.06280365	0.03115941	0.04045815	0.04580381	0.06796599	0
TCGA-XJ-A9DQ-01A	0.03813017	0	0.0513733	0.0193015	0	0
TCGA-YL-A8HO-01A	0.0423393	0.01156509	0	0.03662989	0.30524561	0.05113256
TCGA-ZG-A9MC-01A	0.03677619	0	0.14105364	0.07194275	0.08328419	0
TCGA-XK-AAIV-01A	0	0.02941436	0.16911236	0.08339642	0.11695162	0.03032681
TCGA-ZG-A9KY-01A	0.11914824	0.01121445	0	0.10619257	0.06361413	0.00539883
TCGA-XK-AAIW-01A	0.00505966	0	0.01986174	0.06799822	0.10432552	0.02820281
TCGA-QU-A6IM-01A	0.08558615	0.02623187	0.00596279	0.0415	0.05415695	0.03220102

id	Dendritic cells activated	Mast cells resting	Mast cells activated	Eosinophils	Neutrophils
TCGA-EJ-7794-11A	0	0.12077374	0	0	0.02071842
TCGA-EJ-7783-11A	0.00129233	0.2814893	0	0	0
TCGA-EJ-7330-11A	0.00653706	0.16873975	0.00570031	0	0.0108204
TCGA-EJ-7331-11A	0	0.2112275	0	0	0.01998004
TCGA-EJ-7782-11A	0	0.09206214	0	0	0.00257258
TCGA-EJ-7315-11A	0	0.0912697	0	0	0
TCGA-EJ-7792-11A	0	0.12724833	0	0	0.00847884
TCGA-EJ-7781-11A	0	0.14790041	0	0	0
TCGA-EJ-7328-11A	0	0.1395817	0	0	0
TCGA-EJ-7125-11A	0	0.19685545	0	0.00150193	0
TCGA-HC-7752-11A	0	0.15308836	0	0	0
TCGA-V1-A8WL-01A	0	0.13297226	0	0	0
TCGA-HC-7745-01A	0	0.12726279	0	0.01480999	0
TCGA-V1-A9ZI-01A	0	0.05534854	0	0	0
TCGA-VN-A88I-01A	0.00591849	0.2352678	0	0	0
TCGA-G9-6379-01A	0	0.10813937	0	0	0
TCGA-G9-6347-01A	0.01262964	0.09180206	0	0	0
TCGA-HC-8266-01A	0	0	0.00228452	0	0
TCGA-HC-7233-01A	0	0.06768431	0	0	0.00166578
TCGA-KK-A7B2-01A	0	0.09693298	0	0	0
TCGA-VP-A87C-01A	0	0.04490978	0	0	0
TCGA-J4-A6G1-01A	0.0111694	0.03500091	0	0	0
TCGA-KK-A7AV-01A	0	0.11708052	0	0	0
TCGA-J9-A52E-01A	0	0.0590307	0	0	0
TCGA-EJ-7328-01A	0	0.1051536	0	0	0
TCGA-EJ-7792-01A	0.00299523	0.16450311	0	0	0
TCGA-ZG-A8QW-01A	0	0.04769051	0	0	0
TCGA-EJ-8470-01A	0	0.0211045	0	0	0
TCGA-V1-A8MU-01A	0	0.16573895	0	0	0
TCGA-KK-A6E3-01A	0	0.16231243	0	0	0.00150967
TCGA-4L-AA1F-01A	0	0.04139556	0	0	0
TCGA-EJ-5531-01A	0.01316248	0.10540379	0	0	0
TCGA-ZG-A9LS-01A	0.00583799	0.07589855	0	0	0
TCGA-CH-5754-01A	0	0.06288632	0	0	0
TCGA-J9-A8CP-01A	0.00566721	0.49695743	0	0	0
TCGA-ZG-A9M4-01A	0	0.01626571	0	0	0
TCGA-M7-A723-01A	0	0.08467815	0	0	0
TCGA-KK-A8I4-01A	0	0.13757196	0	0	0
TCGA-EJ-A7NM-01A	0	0.05758833	0	0	0
TCGA-V1-A9O9-01A	0	0.02329374	0	0	0.00245357
TCGA-YL-A9WY-01A	0	0.04932324	0	0	0
TCGA-KK-A59V-01A	0	0.02488729	0	0	0
TCGA-V1-A8MF-01A	0	0.25761385	0	0	0
TCGA-EJ-AB20-01A	0.01007544	0.02811038	0.02070835	0	0
TCGA-ZG-A9N3-01A	0.02596731	0.02112274	0	0	0
TCGA-EJ-A6RC-01A	0	0.07891693	0	0	0
TCGA-XJ-A9DQ-01A	0	0.17070923	0	0	0
TCGA-YL-A8HO-01A	0	0.06893738	0	0	0
TCGA-ZG-A9MC-01A	0.04887408	0.03196478	0	0	0
TCGA-XK-AAIV-01A	0	0.04633761	0	0	0
TCGA-ZG-A9KY-01A	0	0.07880431	0	0	0
TCGA-XK-AAIW-01A	0	0.02657156	0	0	0
TCGA-QU-A6IM-01A	0.00421889	0.03191891	0	0	0

id	B cells naive	B cells memory	Plasma cells	T cells CD8	T cells CD4 naive
TCGA-HC-7817-01B	0	0	0.116025749	0.107950348	0
TCGA-G9-6329-01A	0.036873831	0	0.022188077	0.192118549	0
TCGA-HC-7747-01A	0.034867759	0	0.081620313	0.035793513	0
TCGA-XJ-A9DI-01A	0.044372681	0	0.003400161	0.220973963	0
TCGA-V1-A9Z8-01A	0.001855982	0	0.099703917	0.036476389	0
TCGA-EJ-5510-01A	0.004229867	0	0.133140015	0.129142447	0
TCGA-KK-A7AW-01A	0.010816988	0	0.183465239	0.152584741	0
TCGA-J4-A6G3-01A	0.008698326	0.000388623	0.050131458	0.11210417	0
TCGA-J9-A52C-01A	0.064944534	0	0.038484423	0.153285717	0
TCGA-G9-6498-01A	0.062790493	0	0.095885081	0.17370261	0
TCGA-V1-A9O5-01A	0.036111744	0	0	0.037032948	0
TCGA-J4-A67M-01A	0.004008806	0	0.053359466	0.149176586	0
TCGA-2A-AAYO-01A	0.076958371	0.019835396	0.090841849	0.058728212	0
TCGA-KK-A8IL-01A	0.022642322	0	0.076631448	0.208647011	0
TCGA-J4-A67N-01A	0.012355884	0	0.206360035	0.066739823	0
TCGA-FC-A8O0-01A	0.021374462	0	0.053346868	0.02623144	0
TCGA-CH-5792-01A	0.050649123	0	0.082618915	0.077920877	0
TCGA-CH-5751-01A	0.119912485	0.003218599	0.063807877	0.255898477	0
TCGA-CH-5761-01A	0.023861826	0	0.067394352	0.001373375	0
TCGA-HC-A6AS-01A	0.003137696	0	0.083216201	0.098453349	0
TCGA-KK-A7B0-01A	0.017594518	0	0.09786389	0.072096344	0
TCGA-HC-7079-01A	0.04431272	0.054598716	0.109511072	0.065914395	0
TCGA-ZG-A9LN-01A	0.046059911	0.019344071	0.013965604	0.255428396	0
TCGA-G9-7523-01A	0.020114257	0	0.006626755	0.032422286	0
TCGA-H9-A6BX-01A	0.006385227	0	0.030463705	0.059363932	0
TCGA-EJ-A65B-01A	0	0.00552207	0.26274125	0.030357913	0
TCGA-HC-A9TH-01A	0.022098826	0	0.083007141	0.025725533	0
TCGA-HC-8258-01B	0.079629997	0	0.02239527	0.023949243	0
TCGA-EJ-5509-01A	0.02267401	0	0.033607797	0.073237836	0
TCGA-HC-A9TE-01A	0.0389291	0	0.111001883	0.08942719	0
TCGA-G9-7521-01A	0.026639423	0	0	0.047137704	0

id	T cells CD4 memory resting	T cells CD4 memory activated	T cells follicular helper	T cells regulatory (Tregs)	T cells gamma delta	NK cells resting
TCGA-HC-7817-01B	0.26060032	0.02403738	0.02251027	0	0	0
TCGA-G9-6329-01A	0.29831213	0	0.01998418	0	0.00698124	0
TCGA-HC-7747-01A	0.15681788	0	0.02384466	0	0.03879282	0
TCGA-XJ-A9DI-01A	0.19629212	0	0.02706508	0.03389463	0.01787992	0.0031662
TCGA-V1-A9Z8-01A	0.29630167	0	0	0.0106282	0	0
TCGA-EJ-5510-01A	0.25871222	0	0.04177173	0.00571822	0.0090943	0
TCGA-KK-A7AW-01A	0.17547967	0	0.03038903	0.05348754	0	0
TCGA-J4-A6G3-01A	0.34444216	0.00327545	0.02756301	0.03160265	0	0
TCGA-J9-A52C-01A	0.19225665	0	0.04358194	0.00051535	0.02452261	0
TCGA-G9-6498-01A	0.28825577	0.01866552	0.00758781	0.11810196	0	0
TCGA-V1-A9O5-01A	0.36632246	0	0	0.03638574	0	0.00933293
TCGA-J4-A67M-01A	0.31438426	0.03184713	0	0.04065237	0	0
TCGA-2A-AAYO-01A	0.20951329	0	0.08400984	0.03243629	0.01302316	0
TCGA-KK-A8IL-01A	0.27122489	0.04372546	0.02166292	0.01485017	0	0
TCGA-J4-A67N-01A	0.10494018	0.01296525	0.0145744	0.00799765	0.00137659	0
TCGA-FC-A8O0-01A	0.13557268	0	0.07152365	0.00517989	0	0
TCGA-CH-5792-01A	0.26376605	0	0.05426238	0.04969257	0	0
TCGA-CH-5751-01A	0.19698506	0.011939	0.01736824	0.07509005	0.0123411	0
TCGA-CH-5761-01A	0.0723056	0	0.04636555	0.01444739	0	0
TCGA-HC-A6AS-01A	0.16119401	0.00521129	0.03052433	0.04323665	0	0
TCGA-KK-A7B0-01A	0.29995148	0	0.01550891	0.06838415	0	0
TCGA-HC-7079-01A	0.18590651	0.01051067	0.08251919	0.06441418	0	0.00287787
TCGA-ZG-A9LN-01A	0.12600173	0.01441534	0.06761716	0.09403368	0.01482004	0
TCGA-G9-7523-01A	0.38439627	0	0.01361403	0.04508752	0.01817459	0
TCGA-H9-A6BX-01A	0.25469979	0	0.01249847	0.01719974	0	0
TCGA-EJ-A65B-01A	0.09351067	0	0.00892139	0.00041199	0	0
TCGA-HC-A9TH-01A	0.26984586	0	0.02219916	0.03321962	0.00553896	0
TCGA-HC-8258-01B	0.55927868	0.03825807	0	0	0	0.0236217
TCGA-EJ-5509-01A	0.11773684	0	0.01961409	0.03111792	0	0
TCGA-HC-A9TE-01A	0.313348	0	0.03839619	0.04140897	0	0
TCGA-G9-7521-01A	0.51357375	0.01763135	0	0.00941653	0	0

id	NK cells activated	Monocytes	Macrophages M0	Macrophages M1	Macrophages M2	Dendritic cells resting
TCGA-HC-7817-01B	0.03008989	0.0174628	0.08439592	0.04605038	0.12777043	0.07979912
TCGA-G9-6329-01A	0.04025748	0	0.01334252	0.1082924	0.09025243	0.03158987
TCGA-HC-7747-01A	0.077201	0.01578403	0.07100796	0.17803757	0.0833463	0.06100152
TCGA-XJ-A9DI-01A	0	0	0.16276793	0.11595837	0.12004784	0.02313732
TCGA-V1-A9Z8-01A	0.08254826	0.01427456	0.04020164	0.04948281	0.10297412	0.01736954
TCGA-EJ-5510-01A	0.03979454	0	0.0123232	0.09599109	0.03838749	0.05888455
TCGA-KK-A7AW-01A	0.0642053	0.02142263	0.09844307	0.05308701	0.06729	0.00479897
TCGA-J4-A6G3-01A	0.0395202	0.00303709	0.03869136	0.10953517	0.08874039	0.06424771
TCGA-J9-A52C-01A	0.03304143	0	0.11937894	0.08465943	0.14570068	0.04615134
TCGA-G9-6498-01A	0.02444477	0	0.03856121	0.02889297	0.06521254	0
TCGA-V1-A9O5-01A	0	0.02241528	0.03901142	0.19281294	0.14082597	0.01652061
TCGA-J4-A67M-01A	0.01581987	0.05256423	0.06021918	0.01458771	0.10888558	0.02063106
TCGA-2A-AAYO-01A	0.03143643	0.00595662	0.08775538	0.07044092	0.06831089	0
TCGA-KK-A8IL-01A	0.00154996	0.01885956	0.01449914	0.11168111	0.0961228	0.04844195
TCGA-J4-A67N-01A	0.03387572	0	0.38022661	0.02598743	0.08950942	0.01370529
TCGA-FC-A8O0-01A	0.087167	0.00874198	0	0.04595397	0.11379655	0
TCGA-CH-5792-01A	0.02080798	0.03469195	0.02207552	0.11899101	0.14854428	0.02584064
TCGA-CH-5751-01A	0	0	0.08257363	0.07329329	0.07922439	0
TCGA-CH-5761-01A	0.01352751	0	0.43888964	0.05824479	0.21032238	0.00191553
TCGA-HC-A6AS-01A	0.01960528	0.03014666	0.22074515	0.0410651	0.15795904	0.00545994
TCGA-KK-A7B0-01A	0.03643289	0	0.15068492	0.02448118	0.11878037	0.00351171
TCGA-HC-7079-01A	0	0	0.21513587	0.09175541	0.0716183	0
TCGA-ZG-A9LN-01A	0.04207104	0	0.03742562	0.10516291	0.03165239	0.05427765
TCGA-G9-7523-01A	0.04353307	0.00683956	0.05664451	0.0477486	0.17325005	0.01769289
TCGA-H9-A6BX-01A	0.02813908	0	0.29079994	0.05042307	0.08693165	0.0162847
TCGA-EJ-A65B-01A	0.05865755	0	0.2520037	0.00985092	0.11549811	0.01031955
TCGA-HC-A9TH-01A	0.03680695	0	0.11876511	0.16070557	0.10795443	0.03874219
TCGA-HC-8258-01B	0	0.02621378	0	0.08399031	0.03671079	0
TCGA-EJ-5509-01A	0.03678055	0	0.34307177	0.05179365	0.09047847	0.04401329
TCGA-HC-A9TE-01A	0.02927553	0.00443385	0.0588131	0.06137032	0.08172269	0.03593492
TCGA-G9-7521-01A	0	0.02140678	0.01779109	0.19092375	0.02559532	0.005856

id	Dendritic cells activated	Mast cells resting	Mast cells activated	Eosinophils	Neutrophils
TCGA-HC-7817-01B	0.00686109	0.07062305	0	0	0.00582326
TCGA-G9-6329-01A	0	0.1398073	0	0	0
TCGA-HC-7747-01A	0	0.14188468	0	0	0
TCGA-XJ-A9DI-01A	0	0.03104379	0	0	0
TCGA-V1-A9Z8-01A	0	0.24818291	0	0	0
TCGA-EJ-5510-01A	0	0.17281035	0	0	0
TCGA-KK-A7AW-01A	0	0.08452983	0	0	0
TCGA-J4-A6G3-01A	0	0.07802224	0	0	0
TCGA-J9-A52C-01A	0	0.02111423	0.03236273	0	0
TCGA-G9-6498-01A	0.03523366	0.04266562	0	0	0
TCGA-V1-A9O5-01A	0	0.10322796	0	0	0
TCGA-J4-A67M-01A	0.00805259	0.12581116	0	0	0
TCGA-2A-AAYO-01A	0	0.15075337	0	0	0
TCGA-KK-A8IL-01A	0.01420569	0.03525559	0	0	0
TCGA-J4-A67N-01A	0	0.02938574	0	0	0
TCGA-FC-A8O0-01A	0.00245424	0.42865727	0	0	0
TCGA-CH-5792-01A	0	0.05013872	0	0	0
TCGA-CH-5751-01A	0	0.00834782	0	0	0
TCGA-CH-5761-01A	0	0.05135205	0	0	0
TCGA-HC-A6AS-01A	0	0.10004531	0	0	0
TCGA-KK-A7B0-01A	0	0.09470964	0	0	0
TCGA-HC-7079-01A	0	0.00092509	0	0	0
TCGA-ZG-A9LN-01A	0.02628259	0.04949686	0	0	0.00194501
TCGA-G9-7523-01A	0	0.13385563	0	0	0
TCGA-H9-A6BX-01A	0	0.14681071	0	0	0
TCGA-EJ-A65B-01A	0.02174592	0.13045896	0	0	0
TCGA-HC-A9TH-01A	0	0.07539065	0	0	0
TCGA-HC-8258-01B	0.07302978	0.01372233	0	0.01920005	0
TCGA-EJ-5509-01A	0	0.13587379	0	0	0
TCGA-HC-A9TE-01A	0.0380219	0.05791636	0	0	0
TCGA-G9-7521-01A	0	0.12402829	0	0	0

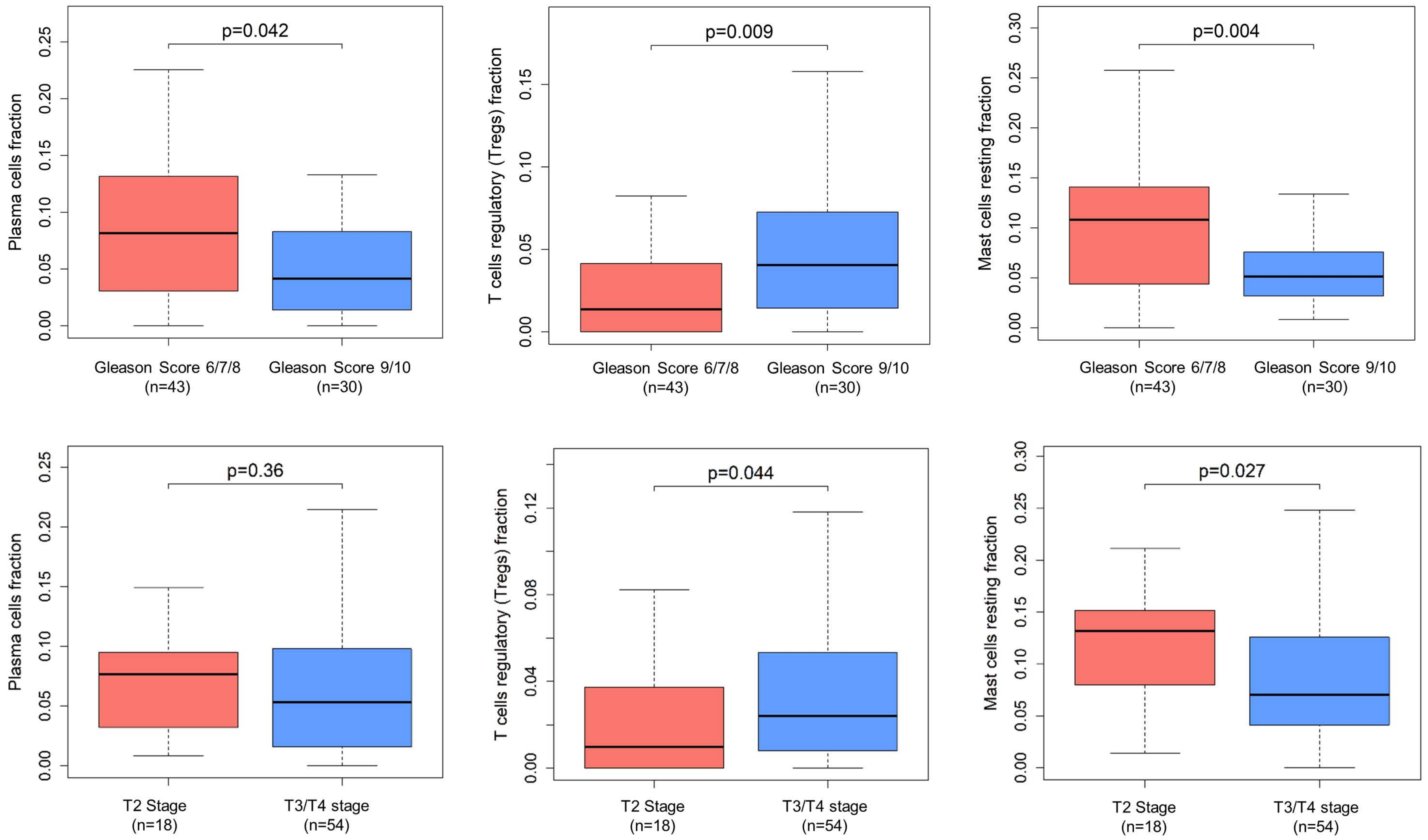

Figure S1. Immune infiltration in different clinical stages.

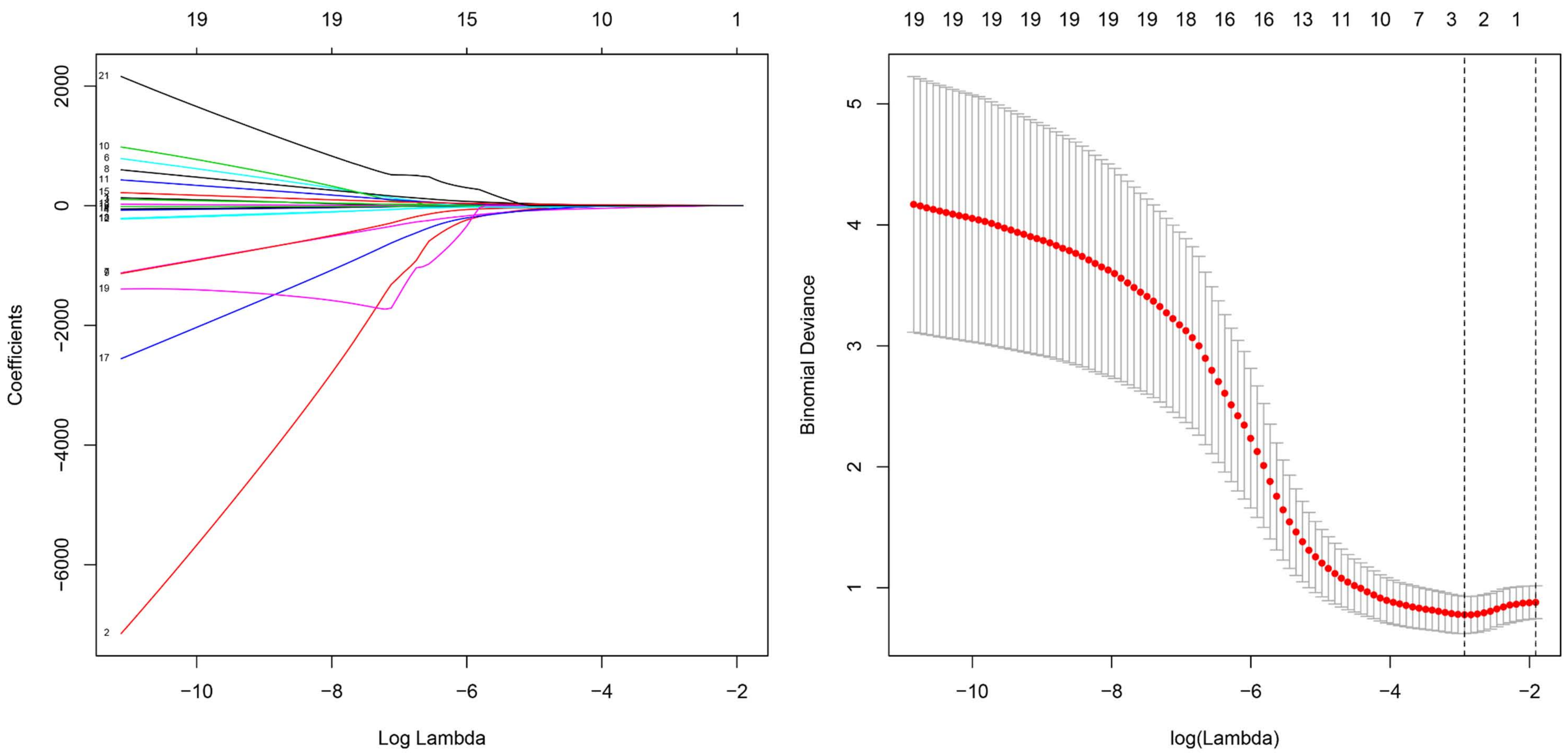

Figure S2 Recurrence associated immune cells selection using the LASSO binary logistic regression model. (A) Optimal parameter (lambda) selection in the LASSO model used fivefold cross-validation via minimum criteria.17,24 The partial likelihood deviance (binomial deviance) curve was plotted versus log(lambda). Dotted vertical lines were drawn at the optimal values by using the minimum criteria and the 1 SE of the minimum criteria (the 1-SE criteria). (B) LASSO coefficient profiles of the 22 features. A coefficient profile plot was produced against the log(lambda) sequence. Vertical line was drawn at the value selected using fivefold cross-validation, where optimal lambda resulted in five features with nonzero coefficients.

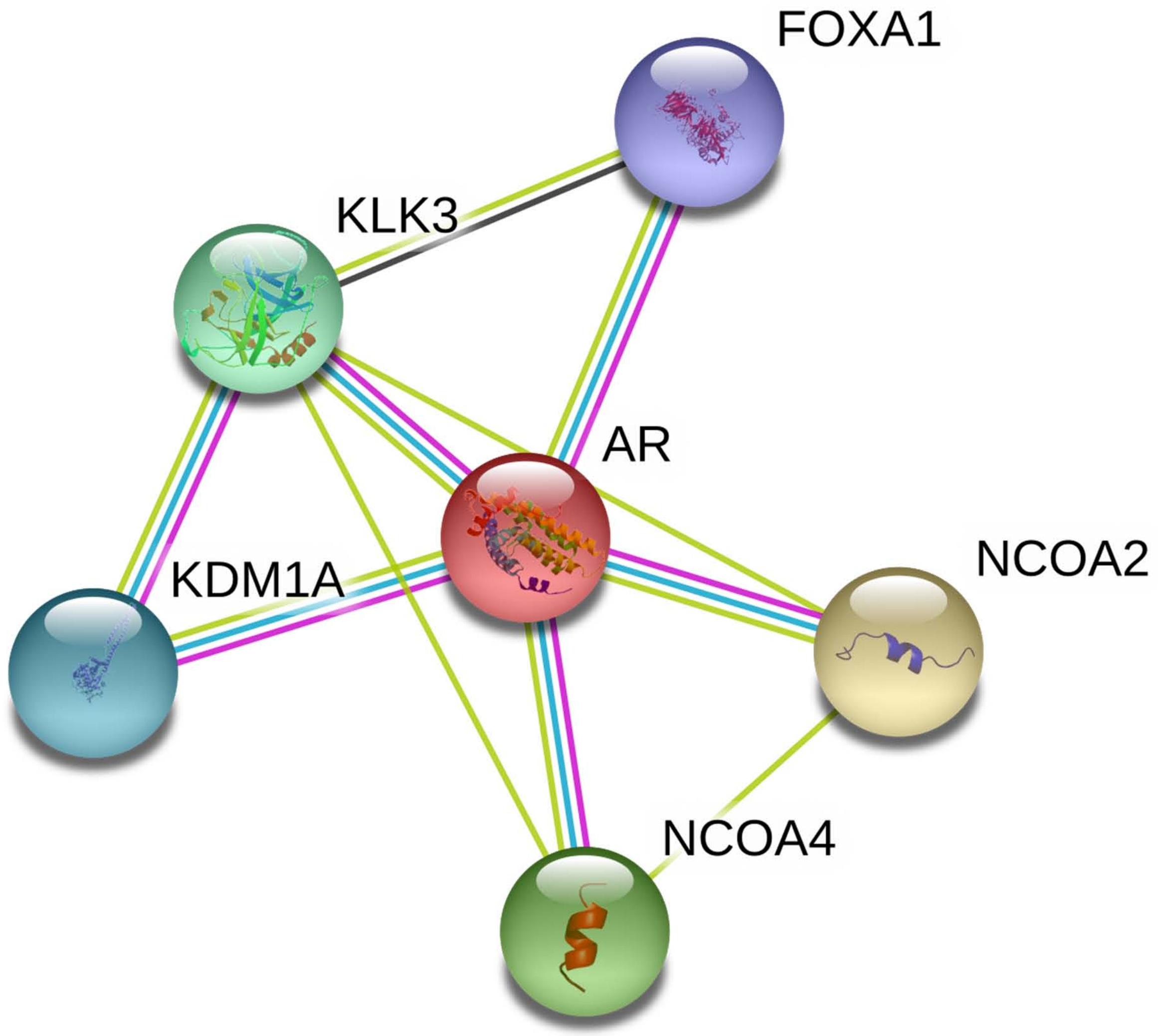

Figure S3. **Top 5 AR related genes.** STRING was employed to reveal the functional protein association networks.

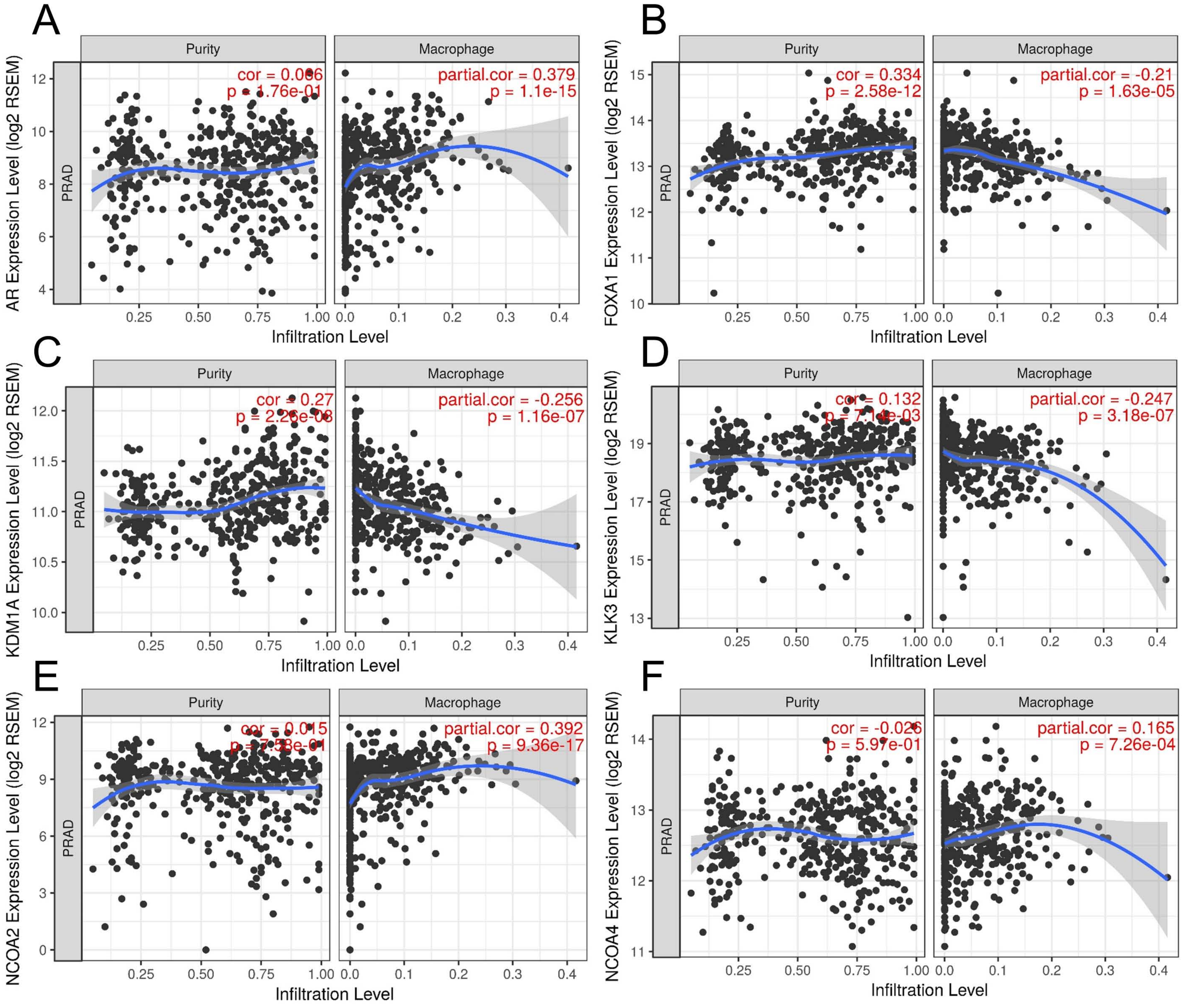

Figure S4 Macrophages infiltration and key genes in PCa. TIMER was managed to evaluate the association between macrophage proportion and key genes in PCa, including AR (A), FOXA1 (B), KDM1A (C), KLK3 (D), NCOA2 (E), NCOA4 (F).
